# Academic and professional profile and impact of graduates from the Nephrology Graduate Program at UNIFESP

**DOI:** 10.1590/2175-8239-JBN-2024-0178en

**Published:** 2025-07-11

**Authors:** Pablo Ferraz, Nestor Schor, Gianna Mastroianni Kirsztajn

**Affiliations:** 1 Universidade Federal de São Paulo, Escola Paulista de Medicina, Fundação Oswaldo Ramos, Disciplina de Nefrologia, São Paulo, SP, Brazil.

**Keywords:** Research, Staff Development, Students, Nephrology, Education, Medi­cal, Graduate, Employment, Academic dissertations as topic.

## Abstract

**Introduction:**

Graduate studies in Brazil have experienced significant growth since the 1990s. Over a 40-year period, the Graduate Program in Nephrology at Unifesp has qualified 261 master’s graduates, 111 doctors and 146 individuals who completed both a master’s and a doctoral degree. Of these, 278 hold a degree in Medicine. Medical postgraduates were responsible for 124 master’s dissertations and 243 doctoral theses completed.

**Objective:**

This study analyzed the profile and professional trajectories of graduate students from the Nephrology Graduate Program at Unifesp.

**Methods:**

The authors used the university’s database to establish the graduates’ profile and applied a questionnaire to identify their professional performance in academia and the job market. The graduates were divided into three groups: G1 – 1976 to 1997 (N = 127); G2 – 1998 to 2006 (N = 150); G3 – 2007 to 2015 (N = 241).

**Results:**

Regarding sex, male medical graduates were responsible for 53.6% of all completion papers; however, in the most recent period, women accounted for 61% of the works. Female participation was consistently higher among graduates from other areas, at 73.8% of the total. Among the physicians, 65.5% graduated from public universities, with the first group standing out with 73%. In the other groups, 59.5% and 59.8% came from public HEIs, respectively. The overall average income reported by master’s graduates responding to the questionnaire ranged from 5 to 10 minimum wages (MW), and for doctors, above 10 MW.

**Conclusion:**

Doctoral graduates had a strong presence in academia, predominantly within the public sector.

## Introduction

Graduate studies in Brazil were formally regulated in 1965, in response to the need to develop a system of education aimed at training researchers and faculty for higher education courses. This system was designed not only to transmit acquired knowledge (as in undergraduate education), but also “to develop new knowledge through creative research activity”^1,2^.

The model adopted for the implementation of graduate programs in Brazil was based on the US system, comprising two successive cycles equivalent to the degrees of *master* and *doctor*. The US model, in turn, was inspired by the German system, which focused on scientific and technological research activities”^1^. Thus, in 1965, Brazil established 27 master’s and 11 doctoral courses, totaling 38 across the country^2^.

By 1998, the year in which Capes – the federal agency responsible for higher education in Brazil – restructured the graduate evaluation system, there were 749 master’s and doctoral programs, 464 master’s courses and 24 exclusively doctoral. Currently, the country has 2,390 graduate programs, including 1,319 master’s and 808 doctoral programs^3^.

Founded in 1933, the *Escola Paulista de Medicina* (EPM) was officially recognized in 1938 and became a federal institution in 1956. Graduate studies associated with research have been present since the early years of the EPM, with the first doctoral thesis defended in 1939. The first formal graduate programs (Molecular Biology, Pharmacology, Histology, Microbiology, and Immunology) were accredited by Capes in early 1970. In 1994, EPM was incorporated into the *Universidade Federal de São Paulo* (Unifesp), which, by 2022, had reached a total of 64 graduate programs recognized by Capes.

The Graduate Program in Nephrology (PPG-N, for its acronym in Portuguese) was established and accredited by the Ministry of Education (MEC), at master’s and doctoral level, in 1973. It was the first course in the area to be regulated in Brazil, becoming one of the most prestigious programs in the country.

In its first 20 years of history, the program awarded master’s and doctoral degrees solely to physicians from medical schools and universities across all five regions of the country. This characteristic has made the PPG-N a program of recognized importance in the training of medical professors and researchers who have gone on to work at their home universities, at other higher education institutions in Brazil, or at our own institution – following the integration of these professionals – in addition to working in other areas of activity.

## Methods

This is a retrospective study, divided into two phases, which analyzed the profile and professional trajectory of graduates from the PPG-N at Unifesp:

**1st phase**: analysis of the profile of the 278 medical postgraduate students and the 367 theses and dissertations they produced between 1976 and 2015. The graduates were divided into three groups: G1 – 1976 to 1997 (N = 127); G2 – 1998 to 2006 (N = 150); and G3 – 2007 to 2015 (N = 241). The first group corresponded to a longer time span, aiming to increase the number of participants so as to make its composition comparable to the other groups. In addition, the decision to start the second group in 1998 was also due to the adoption, in that same year, of excellence indicators by Capes for the evaluation of graduate programs.

The profile information was obtained from the Unifesp Office of Graduate Studies and Research database: level, sex, initial age (at admission) and age at completion, type of Higher Education Institution (HEI) of origin (public or private), and state/country of the HEI.

**2nd phase**: analysis of the responses to the Graduates Questionnaire.

The main tool for evaluating the professional trajectory of graduates was a questionnaire divided into seven modules, consisting of 59 open- and closed-ended questions addressing the academic and professional impact following the completion of graduate studies.

The questionnaire, developed using the Google Forms® platform, was based on Fiocruz’s experience in evaluating graduates, as well as on a study by the Organization for Economic Cooperation and Development (OECD), aimed at mapping the qualifications and mobility of doctors around the world^4,5^.

A pilot version of the questionnaire was administered to 40 randomly selected graduates, in order to validate the questions. Over a three-month period, we obtained an 82.5% response rate (33), a figure higher than that typically observed in online surveys, which averages around 20%^6^. After data tabulation, a panel composed of the author of this study, his primary advisor, and a researcher from PPG analyzed and classified the relevance and importance of the questions using the Likert Scale^7^.

**Figura 1:**
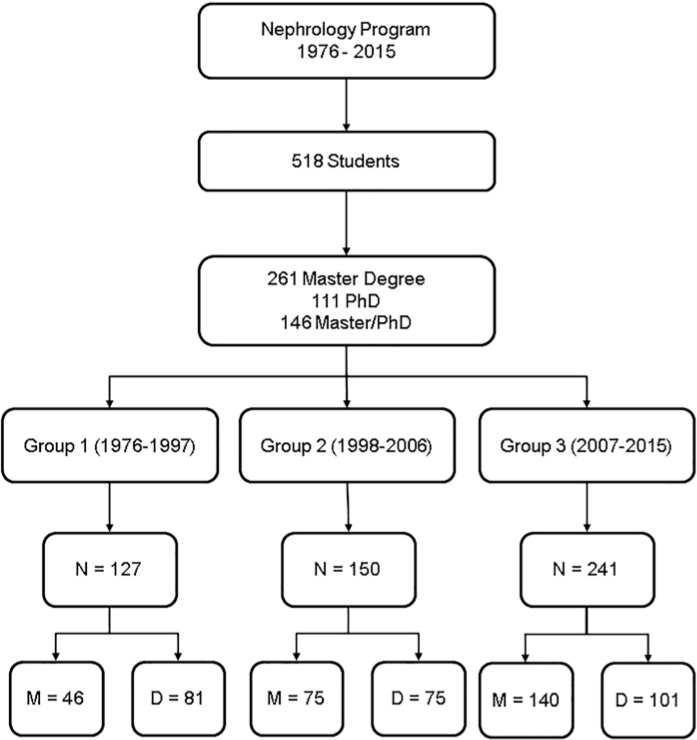
Distribution of questionnaires among graduates and frequencies (%) of sent and responded questionnaires for master's and doctoral degrees.

During the 12-month period from October 2016 to September 2017, the final questionnaire was sent via email to all the graduates whose email addresses were available. Access to the questionnaire was provided through an individual link included in each message. By the end of the questionnaire application period, we had reached 91% (472) of the 518 students and obtained 60% (283) of responses, a rate twice as high as those obtained in similar studies (Figure 1).

## Results

Medical postgraduates were responsible for 124 master’s dissertations and 243 doctoral theses completed in the program within the evaluated period. In the first group, doctoral theses accounted for 65.6%, decreasing to 50% in the second, and 45.1% in the third one. In comparison, graduates from other areas completed 137 master’s dissertations and 160 doctoral theses, with doctoral work representing 66.7% and 64.2% in groups 1 and 2, respectively. In the third group, master’s dissertations accounted for 53.1% of the completion papers.

Male medical graduates authored 53.6% of the theses, with the first period being particularly noteworthy, when they produced 65.6% of the total. However, in the last period, women took the lead, authoring 61% of the theses. Among graduates from other areas, female participation has always been predominant, reaching 73.8%.

Regarding the HEI from which physicians obtained their degree, 65.5% graduated from public institutions, with the first group standing out with 73%. In the other groups, 59.5% and 59.8% were graduates from public HEIs, respectively. Private/confessional institutions were the origin of 70.4% of graduates from other areas, although in the first group, public institutions were the majority (60%). In the second period, the public sector was overtaken by the private one, with 64.5%, reaching 70.4% in the last evaluated period.

The information on the federative region or country of the home HEI revealed that although there was a certain concentration in the Southeast region, with 64.4%, this was considerably lower than that observed among non-medical graduates, for whom the concentration was as high as 90%. For physicians, the variations over the three periods were more similar: 67.2% in G1, 59.5% in G2, and 65.6% in G3. In the case of graduates from other areas, there was an increase from 60% in the first group to 92.5% in the last. It is important to note the presence of medical graduates from the Northeast region, with an average of 15.5%.

An important part of this study was the search for information on the incomes amongst our graduates. The overall average income of master’s graduates who responded to the questionnaire ranged from 5 to 10 Brazilian minimum wages (MW), whereas doctoral graduates reported an income of more than 10 MW. The data by group show that master’s graduates in the first two groups had incomes above 10 MW, while those in the third group had incomes between 5 and 10 MW. Doctoral graduates in G1 were the only group with an average income exceeding 20 MW, since those in the second group earned more than 10 MW on average, and those in the last group, between 5 and 10 MW.

Considering master’s graduates, 40% of women reported incomes above 10 MW, with 15% earning more than 20 MW. Conversely, more than half of the male respondents reported earning more than 10 MW (52%), and 41% of them earned over 20 MW.

We found income disparities between public and private HEI graduates: 38% of those from public HEIs and 16% of those from private HEIs earned more than 20 MW. Of the medical graduates, 88% reported earning more than 10 MW, with 61% receiving over 20 MW. With regard to graduates from other areas, 16% earned more than 10 MW and none of the graduates at this level had incomes of more than 20 MW.

When assessing doctoral graduates, more than half of the men (51%) earned above 20 MW, compared to only 22% of women. In total, 81% of male respondents had incomes of more than 10 MW, compared to 57% of female respondents. This difference is also seen when comparing graduates from public and private HEIs: 46% of public HEI graduates and 22% of private HEI graduates earned more than 20 MW. Only 3% of medical graduates with a doctorate reported incomes of less than 10 MW, while 59% earned more than 20 MW. Among those from other areas, 37% reported earning more than 10 MW, with 10% declaring incomes above 20 MW.

In the master’s program, 47% of graduates were employed at HEIs, while in the doctoral program, this percentage was 66%. The predominant type of employment relationship for the main occupation was governed by Brazil’s Consolidation of Labor Laws (CLT), which accounted for 41% of the total. Those working at public educational institutions, at their different levels (municipal, state, and federal), represented 56% in the first period analyzed, dropping to 27% in the third.

Engagement in teaching and research activities was reported by 81% of doctoral graduates. When analyzing the groups, it was found that 97% of G1 graduates worked in the field. The lowest rate was among the second-period master’s graduates, 53%. Among the reasons cited for choosing to work in teaching or research, specific interest in these areas was pointed out by 43% of the respondents. Even considering the different levels and periods, interest in teaching and research was always the primary reason for professional engagement in these areas.

Of the 283 graduates who responded to the questionnaire, 19% migrated to other states in the country. Of these, 96% were involved in activities related to teaching and research, and 45% had supervised master’s and/or doctoral students through­out their careers.

## Discussion

### Graduate Profile

In 2023, a total of 50,163 master’s degrees and 24,944 doctoral degrees were awarded in the 3,792 graduate programs in Brazil. When compared to the 1998 figures, this represents a 306% increase in the number of master’s graduates (12,351) and a 537% increase in doctoral graduates (3,915) across the 1,259 graduate programs offered at the time^3^. In the USA, according to data from the National Science Foundation, 1,276,394 doctoral degrees were awarded between 1998 and 2023, at an annual growth rate of 1.3%^8^. Information from the same period in Brazil shows that 362,572 doctoral degrees were awarded – 72% lower – but with an annual growth rate of 8.8%^3^.

In the PPG-N, until 1997 – i.e. over 21 years – a total of 109 master’s dissertations and 81 doctoral theses were completed. Between 1998 and 2015, this number increased to 298 master’s dissertations and 176 doctoral theses, corresponding to an increase of 173% and 117%, respectively.

Women represented the majority in the program, accounting for 59% of the finished theses, 64% of the master’s dissertations, and 51% of the doctoral theses. When analyzing only the first period, it was observed that while women participated in 49% of the master’s dissertations, in the doctorate they accounted for only 30% of completions. In G3, women took on a leading role, with 71% of master’s dissertations and 61% of doctoral theses, totaling 67% at both levels.

Between 1996 and 2021, a study by the CGEE (Center for Management and Strategic Studies) revealed that women accounted for 68.9% of all master’s degrees awarded in the broad area of Health Sciences^9^. In the same period, 62.5% of doctoral degrees in this area were awarded to women. A study by Mendes et al.^10^, published in 2010, on graduates of the Health Sciences Program at UFPI, found similar figures, with 68.7% of women completing their master’s degrees. In the USA, between 2000 and 2015, 57.9% of master’s degrees in Biological Sciences were awarded to women; for doctorates, this percentage was 53%^8^.

Our program, which originated from a clinical discipline, began as a postgraduate course almost exclusively aimed at physicians, and by the third period, only 33% of its postgraduates held a degree in Medicine. In Brazil, one of the possible explanations for the decreasing number of physicians in graduate programs is the pursuit of an immediate financial return^11^. In the USA, Rosenberg reports on a number of factors that discourage young physicians from pursuing research careers, including the debt burden resulting from medical school loans, the income disparity between medical practitioners and researchers, and the long training period^12^.

It is also worth highlighting the geographical distribution of Brazilian graduate programs. In 1998, the Southeast region accounted for 61% of the country’s graduate programs. Although, since the late 1990s, there has been some decentralization in the allocation of science and technology resources, in addition to an increase in the number of graduate programs in the other regions of the country, the Southeast still concentrated 43% of the programs in 2022^5^. In that same year, São Paulo state accounted for 20% (914) of Brazil’s graduate programs, while in 1998 this percentage was 36% (450)^5^.

Throughout its history, the PPG-N at Unifesp has received graduates from HEIs in 23 states in all five regions of the country, as well as from abroad. The Southeast region is predominant, contributing with 77% of the students who completed their theses in the program, followed by the Northeast (11%), South (7%), Midwest (3%), and North (2%). Only 1% of students came from abroad.

São Paulo is the state with the most master’s and doctoral degrees in the country. Between 1996 and 2014, 76,212 master’s degrees were awarded, of which 58,725 (77%) graduates were employed in the state in 2014. A total of 58,034 doctoral degrees were issued during this period. Of this total, 31,651 (55%) doctors were employed in São Paulo in that same year.

The state of São Paulo was the origin of 68% of our graduate students and was the destination of 76% of them. Of the graduates from São Paulo, 88% remained in the state, 9% migrated to other states, and 2% moved abroad. Among those from other states, 51% remained in São Paulo, 32% returned to their home states, and 10% relocated elsewhere. A further 7% migrated abroad. This finding confirms the tendency for education and employment of researchers to be concentrated in the Southeast region of Brazil, especially in the state of São Paulo, as also reported by Furtado et al.^13^. Since the inception of our program, efforts have been made to map whether graduates return to their home states, as one of the missions of programs like ours is the eventual nucleation of scholars in their home areas.

Over the entire analyzed period, the average time taken to defend master’s dissertations was 38 months, and 48 months for doctoral theses. Only after the changes in the evaluation system, implemented in 1996^13^ - when Capes began to adopt recommended timeframes of 24 months for master’s degrees and 48 months for doctorates, as criteria for evaluating courses - did the average time for master’s degrees decrease from 46 months in G1 to 34 months in G3.

### Responses to the Questionnaire

Although white people represented 43.5% of the Brazilian population, according to the 2022 Census, graduates who self-declared as white accounted for 80% of all respondents, reaching a total of 91% in the first group. Mixed races, who represented 45.3% of the national population, accounted for 12% of the program’s graduates, followed by Asians, with 6% – a group that represents 1% of Brazil’s general population. Black individuals, who were only present from the second group onwards, accounted for only 1% of graduates, the same percentage as self-declared indigenous people. In the general Brazilian population during this period, blacks accounted for 10.2% and indigenous people for 0.8%^14^. Altogether, black people and mixed races in our program corresponded to 13%, a percentage lower than the 20% reported for graduate programs in Brazil as a whole, drawing particular attention to the fact that only 1% were black^15^.

Among the reasons that led graduates to choose the PPG-N at Unifesp to pursue a master’s degree and/or doctorate, there was a balance between three options: tradition/prestige of the institution (27%), interest in a specific research line (24%), and interest in a particular advisor (24%). However, it is important to note that 51% of the first group preferred the “tradition and prestige” of the university. In the subsequent periods, part of the institution’s prestige was transferred to the program, with 28% of graduates choosing Nephrology due to interest in a specific research line and 25% citing “interest in a particular advisor” as their main motivation for choosing the program. Nevertheless, Unifesp was the main reason for choice for 23% of graduates in groups 2 and 3. Despite the economic downturn of the 1980s and low investment by the Federal Government during the fiscal adjustment of the 1990s, federal public HEIs have remained attractive, regardless of structural problems, partly due to the existence of highly qualified human resources, combined with the expansion of research and graduate studies in the country in the early 2000s^16,17,18^.

It is notable that there was only an increase in the time dedicated to graduate program activities once the program opened to students with degrees in areas other than Medicine. Reports say that medical researchers, in general, do not find the financial and structural incentives to remain in the public teaching and research sphere, which hinders a longer period of dedication. The derisory value of graduate scholarships, the lack of a research career in the country, and the low remuneration for teaching in the public service drive professionals into other areas or reduce their time dedicated to graduate studies^12,13^.

During the period in which medical students were the majority in the program, clinical projects accounted for 47% of all theses. As with the other variables assessed in this study, there was an inversion in the profile of the projects developed in the program – formerly mostly clinical and later predominantly experimental. Among the options for answering this question, there was the possibility of indicating a research project developed in partnership with the private sector – referred to as Industry Interface – which was selected by only 3% of the graduates in the last group. This data may suggest that R&D policies aimed at strengthening innovation in healthcare and creating the conditions for academia to be able to offer a counterpart to society due to the public resources invested, are still incipient in some cases^18^.

Initially, when the majority of students in the program were physicians, research grants did not represent the main source of funding for graduates. However, following the change in the profile of our students, together with the requirement for scholars to dedicate themselves exclusively to postgraduate studies, grants became the primary source of funding for most of them.

Most graduates reported their main employment activity was at a HEI, accounting for 57%. In the master’s program, 47% were employed by HEIs, while in the doctoral program, this percentage was 66% (Table 1).

**Table 1:** Area of final employment of the students who have completed postgraduate

G1 N			G2 N	G3 N	General N
M	Private Sector Company	2 (18%)	6 (21%)	21 (25%)	29 (24%)
	Non-profit Private Sector Company	1 (9%)	5 (17%)	3 (4%)	9 (7%)
	Public Sector Company	1 (9%)	2 (7%)	10 (12%)	13 (11%)
	**Private University**	**1 (9%)**	**4 14%**	**16 (19%)**	**21 (17%)**
	**Public University**	**4 (36%)**	**11 38%**	**20 (24%)**	**35 (28%)**
	Self-employed Professional	2 (18%)	1 3%	13 (16%)	16 (13%)
	Total	11 (100%)	29 (100%)	83 (100%)	123 (100%)
D	Private Sector Company	4 (13%)	4 10%	17 (22%)	25 (16%)
	Non-profit Private Sector Company	1 (3%)	3 7%	5 (6%)	9 (6%)
	Public Sector Company	2 (6%)	3 7%	5 6%	10 (7%)
	**Private University**	**2 (6%)**	**10 24%**	**19 (24%)**	**31 (20%)**
	**Public University**	**22 (69%)**	**21 50%**	**27 (34%)**	**70 (46%)**
	Self-employed Professional	1 (3%)	1 2%	6 (8%)	8 (5%)
	Total	32 (100%)	42 (100%)	79 (100%)	153 (100%)
M/D	Private Sector Company	6 (14%)	10 14%	38 (23%)	54 (20%)
	Non-profit Private Sector Company	2 (5%)	8 11%	8 (5%)	18 (7%)
	Public Sector Company	3 (7%)	5 7%	15 (9%)	23 (8%)
	**Private University**	**3 (7%)**	**14 20%**	**35 (22%)**	**52 (19%)**
	**Public University**	**26 (60%)**	**32 45%**	**47 (29%)**	**105 (38%)**
	Self-employed Professional	3 (7%)	2 3%	19 (12%)	24 (9%)
	Total	43 (100%)	71 (100%)	162 (100%)	276 (100%)

Abbreviations – M: Master degree; D: PhD; M/D: Master degree/PhD.

In June 2016, 96% of the graduates had some form of employment, with 78% of these being formal. When analyzing the groups individually, it was found that 14% of G1 graduates were retired; in G2, this figure was 3%. In G3, 25% of the graduates had no formal employment relationship, and this group also had the highest inactive (unemployed) rate, at 2%. Naturally, the graduates from the earliest analyzed period had the highest number of retired people, while those from the latest period concentrated the largest proportion with no formal employment, due to the shorter time they had been qualified.

In the case of doctorates, our data are in line with those reported by the CGEE, which indicate that the high percentage of newly-graduated doctors with no formal employment relationship may be related to the fact that, to a lesser extent than master’s graduates, these graduates were receiving post-doctoral scholarships at the time the questionnaire was administered, and possibly because one of the main destinations for doctoral graduates is the public university, admission to which occurs through competitive public examinations^9^.

As expected, the salary income of doctoral graduates was higher than that of master’s graduates. Data from the 2014 RAIS (Annual Social Information Report), used in the CGEE study, show that master’s graduates employed in the country earned, on average, R$9,719.00, equivalent to 13.4 MW at the time (R$724.00). Conversely, doctors earned R$13,861.00, or 19 SM on average. The difference in average income between the two academic levels was 29.9%^9^.

In 2014, the average monthly income of women who had obtained a master’s degree since 1996 represented 72.2% of their male counterparts. At doctorate level, the difference between incomes decreased to 85.8%^9^. Compared to our data, in relation to the highest income level (above 20 MW) and considering the entire study period, women earned the equivalent of 74% of what men earned in master’s degrees. Unlike what was observed in the CGEE study, the difference widened at doctoral level, with women earning 71% of men’s income.

According to the CGEE, physicians with a master’s degree since 1996 earned an average of 15 MW by the end of 2014, while professionals from the other courses within the broad area of Health Sciences earned an average of 10 MW^9^. Our data show that 61% of medical graduates at this level earned more than 20 MW, whereas among those from other areas only 16% earned above 10 MW, and none earned more than 20 MW.

At doctorate level, the average income of the physicians monitored by the CGEE was 19 MW compared to 17 MW for the other graduates from the Health Sciences area. Interestingly, the percentage of medical doctors in the program who reported earning more than 20 MW was lower than that of master’s graduates: 59%. However, 10% of graduates from other areas reported income of more than 20 MW. The CGEE also reported a discrepancy between the salaries of health professionals and those from other areas. As the CGEE study relies on official data provided by RAIS, only employer-reported earnings were considered. Given that physicians generally have other employment relationships as self-employed professionals, additional income sources are not captured^9^. In our study, this discrepancy between the educational levels may stem from the fact that physicians with a doctorate are mostly employed at public universities, which require a doctorate degree for admission through competitive examinations, while those with a master’s degree are mostly involved in healthcare activities. In any case, it is also possible to speculate that some of the physicians with doctorates only reported income from their teaching-research activities.

**Figura 2:**
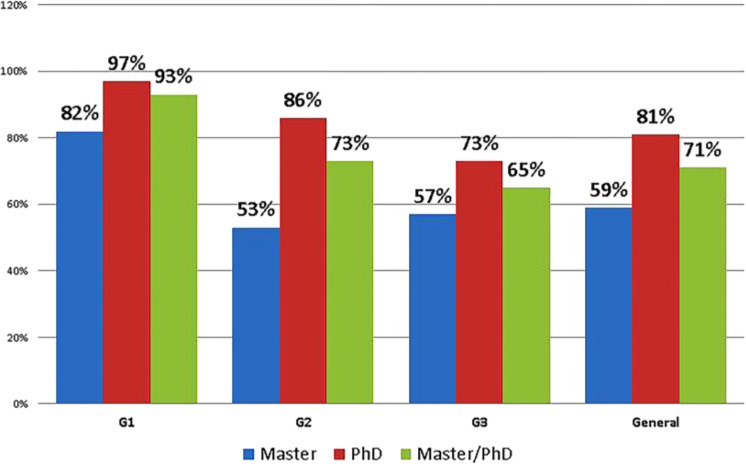
Graduates in teaching-research activities.

Engagement in teaching and research activities was reported by 81% of doctoral graduates. When analyzing the groups, we found that, in G1, activity in the area reached 97%. The lowest rate was observed among master’s graduates from the second period (Figure 2). Unavailability of time or personal reasons were among the main causes for graduates not being involved in research at the time of data collection (42%). The majority of graduates (71%) who were not involved in teaching and/or research expressed their intention to resume working in these areas.

Information on the professional trajectories of graduates from most graduate programs in Brazil is scarce, and even when such data are available, there is no standardized method for data collection or use^18^. This study represents a potential model for continuous research into the situation of postgraduate students in the country. Evaluating the outcome of graduates and their success after completing graduate studies could be one of the ways of measuring the value of existing programs to date, as well as defining which directions graduate studies should take in light of the country’s current reality. In this context, two aspects are considered: meeting minimum needs by providing qualified professors for higher education; or aiming for a situation closer to the ideal, which adds to the preparation of university professors the training of high-level researchers, with prospects of building a respected career and contributing to the development of science and innovation in the country.

We believe that the form/questionnaire used in this study constitutes a proposed tool for mapping graduates, which should preferably be used in an early, standardized, and regular manner after the completion of postgraduate courses.

## Conclusions

The main conclusions drawn at the end of the study were as follows: female participation increased over the periods, and in the most recent one, surpassed that of men. White students predominated, with only 1% of black students. The proportional participation of physicians, in relation to students from other areas, progressively decreased from the first to the last period. The students came from 23 Brazilian states and all five regions of the country, with the state of São Paulo and the Southeast region predominating. The majority (76%) remained in São Paulo state. Initially, most of the students were from public HEIs, but eventually those from private HEIs predominated. We observed that graduate studies had a positive impact on the professional integration of the graduates, with doctoral degrees being associated with higher income levels. Graduates with a doctorate had a strong presence in academia, initially concentrated in the public sector, though a more balanced distribution was observed in the last period. Master’s degree holders, although with a strong presence in universities, migrated to the private sector over the years. The program has contributed to human resource development and the nucleation of new research groups in other states and regions of the country. Notably, 96% of respondents have been engaged in teaching and/or research activities, and 45% have been involved in the training of new master’s and doctoral students across the country.

## Data Availability

Data that are not presented here and corroborate the results of this study will be available upon request to the corresponding author.

## References

[1] Almeida A, Sucupira N, Salgado C, Barreto J, Silva MR, Trigueiro D (2005). Parecer CFE no 977/65, aprovado em 3 dez. 1965.. Presidente da Comissão de Educação Superior. Rev Bras Educ..

[2] Nobre LN, Freitas RR. (2017). A evolução da pós-graduação no Brasil: histórico, políticas e avaliação.. BJPE..

[B3] Capes. (2024). Sistema de Informações Georreferenciadas [Internet]..

[4] Hortale VA, Moreira CO, Bochner R, Leal MC. (2014). Trajetória profissional de egressos de cursos de doutorado nas áreas da saúde e biociências.. Rev Saude Publica..

[5] Auriol L, Schaaper M, Felix M. (2012). Mapping careers and mobility of doctorate holders: draft guidelines, model questionnaire and indicators [Internet].. https://www.oecd.org/content/dam/oecd/en/publications/reports/2012/12/mapping-careers-and-mobility-of-doctorate-holders_g17a2219/5k4dnq2h4n5c-en.pdf.

[B6] Evans JR, Mathur A. (2005). The value of online surveys.. Internet Res..

[7] Likert R. A (1932). technique for the measurement of attitudes. Arch Psychol.

[8] National Center for Science and Engineering Statistics. (2024). Doctorate Recipients from U.S. Universities: 2023. NSF 25-300 [Internet]..

[9] Centro de Gestão e Estudos Estratégicos. (2024). Brasil: Mestres e Doutores 2024..

[10] Mendes FR, Vensceslau EOO, Aires AS, Prado RR (2010). Percepção sobre o curso e perfil dos egressos do Programa de Mestrado em Ciências e Saúde da UFPI.. RBPG..

[11] Carvalho C. (2013). A mercantilização da educação superior brasileira e as estratégias de mercado das instituições lucrativas.. Rev Bras Educ..

[12] Rosenberg L. (1999). Physician-scientists-Endangered and Essential.. Science..

[13] Furtado H, Furtado CA, Davis CA, Gonçalves MA, de Almeida JM. (2015). A spatiotemporal analysis of brazilian science from the perspective of researchers’ career trajectories.. PLoS One..

[14] Instituto Brasileiro de Geografia e Estatística. (2022). Censo Demográfico 2022.

[15] Paixão M, Rossetto I, Montovanele F, Carvano L. (2010). Relatório Anual das Desigualdades Raciais no Brasil.. Rio de Janeiro: Garamond;.

[16] Da Silva AC (2000). Descentralização em política de ciência e tecnologia.. Estud Av..

[17] Soares PC. (2018). Contradições na pesquisa e pós-graduação no Brasil.. Estud Av..

[18] Departamento de Ciência e Tecnologia, Secretaria de Ciência, Tecnologia e Insumos Estratégicos, Ministério da Saúde, Brasilia, Brasil. (2008). Pesquisa em saúde no Brasil.. Rev Saude Publica..

