# Supplementary material for: Academic and professional profile and impact of graduates from the Nephrology Graduate Program at UNIFESP
Source: J Bras Nefrol. 2025 Jul 11;47(3):e20240178. doi: 10.1590/2175-8239-JBN-2024-0178en (PMC12259148; doi:10.1590/2175-8239-JBN-2024-0178en)
Supplement: Translation

# Translation: Perfil e impacto acadêmico e profissional dos egressos do Programa de Pós-Graduação em Nefrologia da UNIFESP

**DOI:** 10.1590/2175-8239-JBN-2024-0178pt

**Authors:** Pablo Ferraz, Nestor Schor, Gianna Mastroianni Kirsztajn

**Affiliations:** 1 Universidade Federal de São Paulo, Escola Paulista de Medicina, Fundação Oswaldo Ramos, Disciplina de Nefrologia, São Paulo, SP, Brasil.

**Keywords:** Pesquisa, Desenvolvimento de Pessoal, Nefrologia, Educação de Pós-Graduação em Medicina, Dissertações acadêmicas como assunto, Aluno, Emprego

## Abstract

**Introdução:**

A pós-graduação no Brasil experimentou crescimento significativo desde os anos 1990. O Programa de Pós-Graduação em Nefrologia da Unifesp formou, ao longo de 40 anos, 261 mestres, 111 doutores e 146 doutores que também realizaram mestrado. Desses, 278 eram graduados em Medicina. Os pós-graduandos médicos foram responsáveis por 124 dissertações de mestrado e 243 teses de doutorado concluídas.

**Objetivo:**

Este estudo analisou perfil e destino dos pós-graduandos no Programa de Pós-Graduação em Nefrologia da Unifesp.

**Métodos:**

Os autores utilizaram o banco de dados da universidade para traçar o perfil dos egressos e aplicaram um questionário para identificar sua atuação profissional no meio acadêmico e no mercado de trabalho. Os egressos foram divididos em três grupos: G1 – 1976 a 1997 (N = 127); G2 – 1998 a 2006 (N = 150); G3 – 2007 a 2015 (N = 241).

**Resultados:**

Quanto ao sexo, os homens formados em Medicina foram responsáveis por 53,6% dos trabalhos de conclusão no total; porém, no último período, as mulheres foram autoras em 61% dos casos. A participação feminina entre os oriundos de outras áreas sempre foi maior, alcançando 73,8% do total. Dos médicos, 65,5% formaram-se em universidades públicas, com destaque para o primeiro grupo, com 73%. Nos demais grupos, 59,5% e 59,8% eram oriundos de IES públicas, respectivamente. A renda média geral dos egressos de mestrado que responderam ao questionário foi de 5 a 10 salários-mínimos (SM) e dos doutores, acima de 10 SM.

**Conclusão:**

Os egressos do doutorado apresentaram forte atuação no meio acadêmico, predominantemente no setor público.

## Introdução

A pós-graduação no Brasil foi regulamentada em 1965, diante da necessidade de se desenvolver no país um sistema de ensino destinado à formação de pesquisadores e docentes para cursos superiores, não apenas focado na transmissão do saber adquirido (graduação), mas também voltado “para a elaboração de novos conhecimentos mediante a atividade de pesquisa criadora”^1,2^.

O modelo seguido para a implantação dos cursos de pós-graduação no Brasil foi o dos EUA, com dois ciclos sucessivos equivalentes aos títulos de *master* e *doctor*. Este, por sua vez, havia sido inspirado no sistema alemão, voltado às atividades de pesquisa científica e tecnológica”^1^. Assim, em 1965, foram criados no Brasil 27 cursos de mestrado e 11 de doutorado, totalizando 38 no país^2^.

Em 1998, ano em que a Capes – órgão federal responsável pelo ensino em nível superior no país – reformulou o sistema de avaliação da pós-graduação, existiam 749 programas de mestrado e doutorado, 464 de mestrado e 24 exclusivamente de doutorado. Atualmente, o país conta com 2.390 programas de mestrado e doutorado, 1.319 somente de mestrado e 808 de doutorado^3^.

Criada em 1933, a Escola Paulista de Medicina (EPM) foi reconhecida oficialmente em 1938 e federalizada em 1956. A pós-graduação associada à pesquisa esteve presente desde os primeiros anos de funcionamento da EPM, com a primeira tese de doutorado defendida em 1939. Os primeiros programas de pós-graduação formais (Biologia Molecular, Farmacologia, Histologia, Microbiologia e Imunologia) foram credenciados pela Capes no início de 1970. Em 1994, a EPM passou a integrar a Universidade Federal de São Paulo (Unifesp), que, em 2022, chegou a ter 64 programas de pós-graduação reconhecidos pela Capes.

O Programa de Pós-Graduação em Nefrologia (PPG-N) foi criado e credenciado pelo MEC para os níveis de mestrado e doutorado em 1973, sendo o primeiro curso na área regulamentado no Brasil e tornando-se um dos programas de maior prestígio no país.

Em seus primeiros 20 anos de história, o programa titulou mestres e doutores exclusivamente médicos, oriundos de escolas médicas e universidades espalhadas pelas cinco regiões do país. Tal característica fez do PPG-N um programa de reconhecida importância na formação de professores e pesquisadores médicos, que passaram a atuar em suas universidades de origem, em outras instituições de ensino superior do país ou em nossa própria instituição – após a absorção desses profissionais – além da atuação em outros ramos de atividade.

## Métodos

Trata-se de um estudo retrospectivo, dividido em duas etapas, que analisou o perfil e o destino dos egressos do PPG-Nefrologia da Unifesp:

**1.ª etapa:** análise do perfil dos 278 alunos médicos de pós-graduação e das 367 teses e dissertações por eles produzidas no período compreendido entre 1976 e 2015. Os egressos foram divididos em três grupos: G1 – 1976 a 1997 (N = 127); G2 – 1998 a 2006 (N = 150); e G3 – 2007 a 2015 (N = 241). O primeiro grupo correspondeu a um maior intervalo de tempo, com o intuito de elevar o número de participantes, de forma a tornar sua composição semelhante à dos demais. Além disso, a escolha de iniciar o segundo grupo em 1998 deveu-se também à adoção, naquele ano, de indicadores de excelência pela Capes para a avaliação dos programas de pós-graduação.

As informações de perfil foram obtidas por meio do banco de dados da Pró-Reitoria de Pós-Graduação e Pesquisa da Unifesp: nível, sexo, idade inicial (na admissão) e de conclusão, tipo de Instituição de Ensino Superior (IES) de origem (pública ou privada) e unidade federativa/país da IES.

**2.ª etapa:** análise das respostas ao Questionário de Egressos.

A principal ferramenta de avaliação do destino dos egressos foi um questionário dividido em sete módulos, composto por 59 perguntas abertas e fechadas sobre o impacto acadêmico e profissional após a conclusão da pós-graduação.

O questionário, desenvolvido a partir da plataforma Formulários Google®, utilizou como base experiências da Fiocruz na avaliação de egressos, bem como um estudo da Organização para a Cooperação e Desenvolvimento Econômico (OECD), voltado ao mapeamento da titulação e mobilidade de doutores em todo o mundo^4,5^.

O questionário piloto foi aplicado a 40 egressos contatados aleatoriamente para validação das perguntas. Em um período de três meses, obtivemos 82,5% de taxa de resposta (33), índice superior ao geralmente observado em pesquisas *online*, que gira em torno de 20%^6^ Após a tabulação dos dados, uma banca composta pelo autor deste estudo, seu primeiro orientador e um pesquisador do PPG analisou e classificou a relevância e importância das questões, de acordo com a Escala de Likert^7^.

Durante o período de 12 meses, que se estendeu de outubro de 2016 a setembro de 2017, o questionário final foi enviado por correio eletrônico a todos os egressos dos quais se dispunha de endereço eletrônico. O acesso se deu por meio de um *link* individual encaminhado em cada mensagem. Ao final do período de aplicação do questionário, alcançamos 91% (472) dos 518 alunos e obtivemos 60% (283) de respostas, uma taxa que representa o dobro daquelas obtidas em estudos similares (Figura 1).

**Figura 1:** Distribuição dos questionários entre os alunos egressos e frequências (%) de envio e de respostas para mestrado e doutorado.

## Resultados

Os pós-graduandos médicos foram responsáveis por 124 dissertações de mestrado e 243 teses de doutorado concluídas no programa dentro do período avaliado. No primeiro grupo, as teses de doutorado representaram 65,6%, passando a 50% no segundo e a 45,1% no terceiro. Em comparação, os formados em outras áreas concluíram 137 dissertações de mestrado e 160 teses de doutorado, sendo que os trabalhos de doutorado representaram 66,7% e 64,2%, nos grupos 1 e 2, respectivamente. No terceiro grupo, as dissertações de mestrado corresponderam a 53,1% dos trabalhos de conclusão.

Os homens formados em Medicina responderam por 53,6% das teses, com destaque para o primeiro período, em que produziram 65,6% dos trabalhos. No entanto, no último período, as mulheres assumiram o protagonismo, sendo autoras de 61% das teses. Já entre os egressos de outras áreas, a participação feminina sempre foi predominante, chegando a 73,8%.

No que diz respeito à IES de origem, 65,5% dos médicos se formaram em instituições públicas, com destaque para o primeiro grupo, com 73%. Nos demais grupos, 59,5% e 59,8% eram oriundos de IES públicas, respectivamente. As privadas/confessionais foram a origem de 70,4% dos egressos formados nas outras áreas, embora, no primeiro grupo, as públicas tenham sido maioria (60%). No segundo período, as públicas foram ultrapassadas pelas privadas, com 64,5%, atingindo 70,4% no último período avaliado.

As informações sobre a região federativa ou país de origem da IES nos mostraram que, embora exista certa concentração na região Sudeste, com 64,4%, ela foi consideravelmente menor do que a verificada no caso dos egressos não médicos, cuja concentração chegou a 90%. Para os médicos, as variações ocorridas ao longo dos três períodos foram mais próximas: 67,2% no G1, 59,5% no G2 e 65,6% no G3. Já no caso dos egressos de outras áreas, houve um salto de 60% no primeiro grupo para 92,5% no último. É importante salientar a presença de egressos médicos oriundos da região Nordeste, com média de 15,5%.

Parte importante deste estudo foi a busca de informações sobre os rendimentos de nossos egressos. A renda média geral dos egressos de mestrado que responderam ao questionário foi de 5 a 10 salários mínimos (SM), enquanto a dos doutores foi superior a 10 SM. Os dados por grupo revelam que os mestres dos dois primeiros grupos tinham rendimentos acima de 10 SM e, no terceiro, entre 5 e 10 SM. Os egressos do doutorado do G1 foram os únicos com renda média maior que 20 SM, uma vez que os do segundo grupo ganhavam mais de 10 SM em média, e os do último grupo, entre 5 e 10 SM.

Considerando os egressos do mestrado**,** o percentual de mulheres com rendimentos acima de 10 SM foi de 40%, sendo que 15% recebiam mais de 20 SM. Por outro lado, mais da metade dos homens que responderam ao questionário declararam receber mais de 10 SM (52%) e 41% deles recebiam acima de 20 SM.

Constatamos diferença salarial entre os formados em IES públicas e privadas: 38% dos oriundos de IES públicas e 16% dos formados em IES privadas recebiam acima de 20 SM. Dos graduados em Medicina, 88% recebiam mais de 10 SM, sendo que 61% ganhavam mais de 20 SM. No que diz respeito aos egressos formados em outras áreas, 16% recebiam mais de 10 SM e não houve egresso nesse nível com rendimentos superiores a 20 SM.

Quando avaliados os egressos do doutorado**,** mais da metade dos homens (51%) recebiam acima de 20 SM, em comparação com apenas 22% das mulheres. No total, 81% dos homens apresentaram rendimentos superiores a 10 SM, contra 57% das mulheres que responderam ao questionário. Tal diferença também é verificada quando comparamos os formados em IES públicas e privadas: 46% dos formados em IES públicas e 22% dos formados em IES privadas recebiam acima de 20 SM. Apenas 3% dos egressos médicos do doutorado tinham renda inferior a 10 SM, enquanto 59% ganhavam mais de 20 SM. Entre os formados em outras áreas, 37% informaram receber mais de 10 SM, sendo que 10% declararam renda superior a 20 SM.

No mestrado, 47% dos egressos estavam empregados em IES, enquanto, no doutorado, esse percentual foi de 66%. O tipo de vínculo predominante na ocupação principal foi o regime CLT, presente em 41% do total. Os trabalhadores vinculados às instituições públicas de ensino, em seus diferentes níveis (municipal, estadual e federal), representaram 56% no primeiro período analisado e passaram a 27% no terceiro.

O envolvimento com atividades de docência e pesquisa foi apontado por 81% dos egressos do doutorado. Observando os grupos, verificou-se que, no G1, a atuação na área alcançou 97%. A menor taxa ocorreu nos egressos do mestrado do segundo período, 53%. Entre os motivos para a escolha de exercer atividade em docência ou pesquisa, o interesse específico nessas áreas foi o apontado por 43% dos respondentes. Mesmo considerando os diferentes níveis e períodos, o interesse por docência e pesquisa sempre foi o principal motivo para a atuação na área.

Dos 283 egressos que responderam ao questionário, 19% migraram para outros estados do país. Desses, 96% estavam envolvidos com atividades relacionadas à docência e pesquisa e 45% formaram mestres e/ou doutores ao longo de sua carreira.

## Discussão

### Perfil do Egresso

Em 2023, foram titulados 50.163 mestres e 24.944 doutores nos 3.792 programas de pós-graduação do Brasil. Ao compararmos esses números aos dados de 1998, constatamos um aumento de 306% no número de mestres formados (12.351) e de 537% no de doutores (3.915), nos 1.259 programas de pós-graduação existentes à época^3^. Nos EUA, de acordo com dados da *National Science Foundation*, entre 1998 e 2023, foram titulados 1.276.394 doutores, a uma taxa de crescimento anual de 1,3%^8^. As informações do mesmo período no Brasil revelam a titulação de 362.572 doutores, 72% a menos, mas com uma taxa de crescimento anual de 8,8%^3^.

No PPG-N, até 1997 - ou seja, em 21 anos - foram concluídas 109 dissertações de mestrado e 81 teses de doutorado. Entre 1998 e 2015, esse número saltou para 298 de mestrado e 176 de doutorado, o que corresponde a um crescimento de 173% e 117%, respectivamente.

As mulheres foram maioria no programa, responsáveis por 59% das teses concluídas, sendo 64% das dissertações de mestrado e 51% das teses de doutorado. Ao analisarmos apenas o primeiro período, observamos que, enquanto no mestrado as mulheres tiveram participação em 49% das dissertações, no doutorado corresponderam a apenas 30% dos concluintes. No G3, as mulheres assumem o protagonismo, com 71% das dissertações de mestrado e 61% das teses de doutorado, perfazendo 67% nos dois níveis.

Entre 1996 e 2021, um estudo do CGEE (Centro de Gestão e Estudos Estratégicos) revelou que as mulheres foram responsáveis por 68,9% dos títulos de mestre na grande área de Ciências da Saúde^9^. No mesmo período, 62,5% dos títulos de doutorado na mesma área foram concedidos a mulheres. Um estudo de Mendes et al*.* ^10^, publicado em 2010, sobre os egressos do Programa em Ciências da Saúde da UFPI, encontrou números semelhantes, com 68,7% de mulheres concluindo o mestrado. Nos EUA, em período próximo no tempo, entre 2000 e 2015, 57,9% dos títulos de mestres na área de *Biological Sciences* foram concedidos a mulheres; no doutorado, esse percentual foi de 53%^8^.

O programa, que teve origem em uma disciplina clínica, iniciou-se como uma pós-graduação quase que exclusivamente voltada para médicos e, no terceiro período, teve somente 33% dos seus pós-graduandos formados em Medicina. No Brasil, tem-se aventado, como uma das explicações para a redução da presença de médicos como alunos de programas de pós-graduação, a busca por retorno financeiro imediato^11^. Nos EUA, Rosenberg relata um conjunto de fatores que afastam o jovem médico da carreira de pesquisador, como o ônus da dívida decorrente do financiamento do curso de Medicina, a disparidade de rendimentos entre os que estão na prática médica e os pesquisadores, além do longo período de formação^12^.

Vale destacar ainda a distribuição geográfica dos programas brasileiros de pós-graduação. A região Sudeste possuía, em 1998, 61% dos programas de pós-graduação do país. Embora, desde o final dos anos 1990, tenha ocorrido certa descentralização na destinação de recursos em ciência e tecnologia, além de um crescimento no número de programas de pós-graduação nas demais regiões do país, o Sudeste ainda concentrava, em 2022, 43% dos programas^5^. Nesse mesmo ano, São Paulo reunia 20% (914) dos programas de pós-graduação do país, enquanto, em 1998, esse percentual era de 36% (450)^5^.

Em toda a sua história, o PPG-N da Unifesp recebeu alunos formados em IES de 23 estados das cinco regiões do país e também do exterior. A região Sudeste é predominante, contribuindo com 77% dos alunos que concluíram suas teses no programa, seguida pela região Nordeste (11%), Sul (7%), Centro-Oeste (3%) e Norte (2%). Do exterior, veio apenas 1% do alunato.

São Paulo é o estado que mais titula mestres e doutores no país. Entre 1996 e 2014, foram titulados 76.212 mestres, dos quais 58.725 (77%) estavam empregados no estado em 2014. Foram emitidos 58.034 títulos de doutorado nesse período. Desse total, 31.651 (55%) doutores estavam empregados em São Paulo no mesmo ano.

O estado de São Paulo foi a origem de 68% dos nossos pós-graduandos e concentrou o destino de 76% deles. Dos egressos com origem de São Paulo, 88% permaneceram no estado, 9% migraram para outras unidades da federação e 2% foram para o exterior. Entre aqueles provenientes de outros estados, 51% permaneceram em São Paulo, 32% voltaram aos seus estados de origem e 10% foram para outras localidades. Ainda, 7% migraram para o exterior. Este achado confirma a tendência de concentração da educação e do emprego dos pesquisadores no Sudeste brasileiro, notadamente no estado de São Paulo, também relatada por Furtado et al.^13^. Desde o início do nosso programa, tem-se buscado mapear se os titulados retornam aos seus estados de origem, pois a eventual nucleação nas áreas de procedência é uma das missões de programas como o nosso.

Em todo o período, o tempo médio para defesa das dissertações de mestrado foi de 38 meses e de 48 meses para as teses de doutorado. Somente após as alterações na sistemática de avaliação, ocorridas em 1996^13^, quando a Capes passou a adotar os prazos recomendáveis de 24 meses para o mestrado e de 48 para o doutorado como quesitos de avaliação dos cursos, o tempo médio no mestrado passou de 46 no G1 para 34 meses no G3.

### Respostas ao Questionário

Embora os brancos correspondessem a 43,5% da população brasileira, de acordo com o Censo 2022, os egressos que se autodeclararam brancos representavam 80% do total dos que responderam ao questionário, tendo alcançado 91% no primeiro grupo. Os pardos, que representavam 45,3% da população nacional, correspondiam a 12% dos egressos do programa, seguidos pelos amarelos, com 6%, grupo que representa 1% da população geral do Brasil. Os pretos, presentes somente a partir do segundo grupo, representavam apenas 1% dos egressos, o mesmo percentual dos autodeclarados indígenas. Na população brasileira, de modo geral, nesse período, os pretos correspondiam a 10,2%, e os indígenas, a 0,8%^14^. Em conjunto, pretos e pardos no nosso programa corresponderam a 13%, um percentual inferior aos 20% relatados para a pós-graduação no Brasil como um todo, chamando particularmente a atenção o fato de que apenas 1% eram pretos^15^.

Entre os motivos que levaram o egresso a escolher o PPG-N da Unifesp para realizar o mestrado e/ou o doutorado, houve equilíbrio entre três opções: tradição/prestígio da instituição (27%), interesse por uma linha de pesquisa específica (24%) e interesse em um orientador específico (24%). Contudo, é importante ressaltar a preferência de 51% do primeiro grupo pela “tradição e prestígio” da universidade. Nos períodos seguintes, houve uma transferência de parte desse prestígio da instituição para o programa, com 28% dos egressos escolhendo a Nefrologia por interesse em uma linha de pesquisa específica e 25% referindo como principal motivo de escolha “o interesse por um orientador específico”. Ainda assim, a Unifesp foi o motivo da escolha para 23% dos egressos nos grupos 2 e 3. Não obstante a retração econômica dos anos 1980 e o baixo investimento por parte da União durante o ajuste fiscal da década de 1990, as IES públicas federais mantiveram-se atrativas, apesar dos problemas estruturais, em parte devido à existência de recursos humanos altamente qualificados, aliada à expansão da pesquisa e da pós-graduação no país no início dos anos 2000^16-18^.

É notório que só houve aumento do tempo de dedicação às atividades da pós-graduação propriamente dita quando o programa passou a permitir o ingresso de alunos formados em outras áreas, além da Medicina. Há relatos de que o médico pesquisador, de um modo geral, não encontra incentivos financeiros e estruturais para se manter na esfera pública de ensino e pesquisa, o que dificulta um maior período de dedicação. Os valores irrisórios das bolsas de pós-graduação, a inexistência de uma carreira de pesquisador no país e a baixa remuneração da atividade docente no serviço público impelem o profissional para outras áreas ou reduzem seu tempo de dedicação à pós-graduação,^12,13^.

Durante o período em que os alunos médicos foram maioria no programa, os projetos clínicos responderam por 47% do total de teses. Assim como ocorreu com outras variáveis avaliadas neste estudo, houve uma inversão no perfil dos projetos desenvolvidos no programa - antes majoritariamente clínicos e, posteriormente, predominantemente experimentais. Entre as opções de resposta para essa questão, havia a possibilidade de indicação de projeto de pesquisa desenvolvido em parceria com a iniciativa privada – Interface com a Indústria – que foi a opção de apenas 3% dos egressos do último grupo. Esse dado pode sugerir que as políticas de P&D que visam fortalecer a inovação em saúde e criar condições para que a academia possa oferecer uma contrapartida à sociedade devido aos recursos públicos investidos, ainda são incipientes em alguns casos^18^.

Inicialmente, quando a maioria dos alunos do programa era formada por médicos, as bolsas de pesquisa não constituíam a principal fonte de recursos dos egressos. No entanto, após a mudança do perfil de nosso aluno em conjunto com a exigência de dedicação exclusiva por parte do bolsista à pós-graduação, as bolsas passaram a representar a principal fonte de financiamento para a maior parte deles.

A maioria dos egressos relatou vínculo principal em IES, totalizando 57%. No mestrado, 47% estão empregados em IES, enquanto, no doutorado, esse percentual é de 66% (Tabela 1).

**Tabela 1:** Setor do empregador do egresso em seu destino

G1 N			G2 N	G3 N	General N
M	Empresa do Setor Privado	2 (18%)	6 (21%)	21 (25%)	29 (24%)
	Empresa do Setor Privado sem fins lucrativos	1 (9%)	5 (17%)	3 (4%)	9 (7%)
	Empresa do Setor Público	1 (9%)	2 (7%)	10 (12%)	13 (11%)
	**Instituição de Ensino Privada**	**1 (9%)**	**4 14%**	**16 (19%)**	**21 (17%)**
	**Instituição de Ensino Pública**	**4 (36%)**	**11 38%**	**20 (24%)**	**35 (28%)**
	Profissional liberal	2 (18%)	1 3%	13 (16%)	16 (13%)
	Total	11 (100%)	29 (100%)	83 (100%)	123 (100%)
D	Empresa do Setor Privado	4 (13%)	4 10%	17 (22%)	25 (16%)
	Empresa do Setor Privado sem fins lucrativos	1 (3%)	3 7%	5 (6%)	9 (6%)
	Empresa do Setor Público	2 (6%)	3 7%	5 6%	10 (7%)
	**Instituição de Ensino Privada**	**2 (6%)**	**10 24%**	**19 (24%)**	**31 (20%)**
	**Instituição de Ensino Pública**	**22 (69%)**	**21 50%**	**27 (34%)**	**70 (46%)**
	Profissional liberal	1 (3%)	1 2%	6 (8%)	8 (5%)
	Total	32 (100%)	42 (100%)	79 (100%)	153 (100%)
M/D	Empresa do Setor Privado	6 (14%)	10 14%	38 (23%)	54 (20%)
	Empresa do Setor Privado sem fins lucrativos	2 (5%)	8 11%	8 (5%)	18 (7%)
	Empresa do Setor Público	3 (7%)	5 7%	15 (9%)	23 (8%)
	**Instituição de Ensino Privada**	**3 (7%)**	**14 20%**	**35 (22%)**	**52 (19%)**
	**Instituição de Ensino Pública**	**26 (60%)**	**32 45%**	**47 (29%)**	**105 (38%)**
	Profissional liberal	3 (7%)	2 3%	19 (12%)	24 (9%)
	Total	43 (100%)	71 (100%)	162 (100%)	276 (100%)

Abreviações – M: Mestrado; D: Doutorado; MD: Mestrado/Doutorado.

Em junho de 2016, 96% dos egressos tinham algum vínculo empregatício, sendo 78% deles formal. Ao analisar individualmente os grupos, verificou-se que, no G1, 14% estavam aposentados; no G2, esse índice foi de 3%. No G3, 25% dos egressos não possuíam vínculo empregatício formal, grupo esse que também apresentou o maior índice de inativos (desempregados), com 2%. Naturalmente, os egressos formados no primeiro período analisado apresentavam o maior contingente de aposentados, enquanto os formados no último período eram os que concentravam a maior parcela sem vínculo formal, em razão do menor tempo de titulação.

No caso do doutorado, nossos dados vão ao encontro dos apresentados pelo CGEE, que indicam que o percentual elevado de recém-doutores sem vínculo formal pode estar relacionado ao fato de, em menor proporção se comparados aos mestres, esses egressos usufruíam de bolsas de pós-doutorado no momento da aplicação do questionário e, possivelmente, porque os doutores formados têm como um dos principais destinos a universidade pública, cujo ingresso ocorre por meio de disputados concursos públicos^9^.

Como esperado, a renda salarial dos egressos de doutorado foi superior à dos egressos do mestrado. Dados da RAIS (Relação Anual de Informações Sociais) de 2014, utilizados no estudo do CGEE, revelam que os mestres empregados no país recebiam, em média, R$ 9.719,00, equivalente a 13,4 SM da época (R$ 724,00). Os doutores, por sua vez, recebiam R$ 13.861,00, ou 19 SM em média. A diferença na renda média entre os dois níveis foi de 29,9%^9^.

Em 2014, a renda média mensal das mulheres que haviam obtido o título de mestre desde 1996 representava 72,2% da renda dos homens. No doutorado, a diferença entre os rendimentos diminuiu, passando a representar 85,8%^3^. Comparando com os nossos dados, em relação ao maior patamar de rendimento (acima de 20 SM) e considerando todo o período, no mestrado as mulheres recebiam o equivalente a 74% do que recebiam os homens. Diferentemente do observado no estudo do CGEE, no doutorado a diferença aumentou, com as mulheres passando a ganhar 71% do rendimento dos homens.

Segundo o CGEE, mestres titulados desde 1996 formados em Medicina recebiam, em média, 15 SM ao final de 2014, enquanto os profissionais oriundos dos demais cursos que compõem a grande área de Ciências da Saúde ganhavam, em média, 10 SM^9^. Nossos dados mostram que 61% dos egressos médicos nesse nível recebiam acima de 20 SM, enquanto, entre os de outras áreas, somente 16% recebiam acima de 10 SM e nenhum acima de 20 SM.

No doutorado, a renda média dos médicos acompanhados pelo CGEE foi de 19 SM e a dos demais titulados provenientes da área de Ciências da Saúde foi de 17 SM. Curiosamente, o percentual de doutores médicos do programa que informaram receber acima de 20 SM foi menor do que a dos mestres: 59%. Porém, 10% dos graduados em outras áreas informaram receber mais de 20 SM. O CGEE também notificou a discrepância entre os salários dos profissionais da área da saúde e os de outras áreas. Como o estudo do CGEE considera dados oficiais fornecidos pela RAIS, apenas os rendimentos informados pelos empregadores foram considerados. Levando-se em conta que médicos, em geral, possuem outros vínculos como profissionais liberais, as rendas adicionais não são captadas^9^. Em nosso trabalho, tal discrepância entre os níveis pode ter origem no fato de que os médicos com doutorado estão, em sua maioria, empregados em universidades públicas, que exigem o título de doutor para ingresso por concurso, enquanto os mestres dedicam-se em sua maioria às atividades assistenciais. De qualquer forma, é possível também especular que parte dos médicos doutores tenha informado somente os rendimentos advindos de suas atividades em docência e pesquisa.

O envolvimento com atividades de docência e pesquisa foi relatado por 81% dos egressos do doutorado. Ao observarmos os grupos, verificamos que, no G1, a atuação na área alcançou 97%. A menor taxa ocorreu nos egressos do mestrado do segundo período (Figura 2). A indisponibilidade de tempo ou motivos pessoais estão entre as principais razões para o egresso não estar envolvido em pesquisa no momento da coleta dos dados (42%). A maioria dos egressos (71%) que se encontrava fora das áreas de docência e/ou pesquisa manifestou intenção de retomar a atuação nessas áreas.

**Figura 2:** Pós-graduandos egressos em atividades de ensino e pesquisa.

São escassas as informações sobre o destino dos egressos na maioria dos programas de pós-graduação no Brasil e, mesmo quando disponíveis, não há padronização quanto à coleta de dados ou de sua utilização^18^. Nosso estudo se constitui como um possível modelo para a realização de pesquisa continuada sobre a situação dos egressos de pós-graduação no país. A avaliação do destino dos egressos e de seu sucesso após a conclusão da pós-graduação pode ser uma das formas de mensurar o valor dos programas existentes até o momento, além de definir quais rumos a pós-graduação deverá seguir diante da realidade do país. Considera-se, nesse contexto, duas vertentes: atender às necessidades mínimas, fornecendo docentes qualificados para o ensino superior; ou almejar situação mais próxima do ideal, que agregue à preparação de professores universitários a formação de pesquisadores de alto nível, com perspectivas de construir uma carreira respeitada e contribuir para o desenvolvimento da ciência e da inovação no país.

Consideramos que o formulário/questionário aqui utilizado se configura como uma proposta de ferramenta para mapeamento de egressos, a ser utilizada preferencialmente de forma precoce, padronizada e regular após a conclusão dos cursos de pós-graduação.

## Conclusões

As principais conclusões a que chegamos ao final do estudo foram: a participação das mulheres cresceu ao longo dos períodos e, no último, foi superior à dos homens. Predominaram alunos brancos, sendo que o percentual de pretos foi de apenas 1%. A participação proporcional de médicos, em relação a alunos de outras áreas, diminuiu progressivamente do primeiro para o último período. Os alunos eram provenientes de 23 estados brasileiros e das cinco regiões do país, com predomínio do estado de São Paulo e da região Sudeste. O destino da maioria deles (76%) foi o próprio estado de São Paulo. Inicialmente, os alunos eram oriundos, sobretudo, de IES públicas, mas ao final predominaram aqueles de IES privadas. Verificamos que a realização da pós-graduação teve impacto positivo na inserção profissional dos egressos, constatando-se que a titulação de doutor associou-se a níveis salariais mais elevados. Os egressos do doutorado tiveram forte atuação no meio acadêmico, inicialmente com predomínio no setor público, mas com equilíbrio no último período, enquanto os mestres, embora com importante atuação nas universidades, migraram ao longo dos anos para o setor privado. O programa contribuiu para a formação de recursos humanos e nucleação de novos grupos de pesquisa em outros estados e regiões do país, observando-se que 96% deles envolveram-se com atividades de docência e/ou pesquisa e 45% atuaram na formação de mestres e doutores pelo país.

## Data Availability

Os dados que não se encontram aqui expostos e corroboram os resultados deste estudo estarão disponíveis mediante solicitação ao autor correspondente.
